# NeuroMEMS: Neural Probe Microtechnologies

**DOI:** 10.3390/s8106704

**Published:** 2008-10-25

**Authors:** Mohamad HajjHassan, Vamsy Chodavarapu, Sam Musallam

**Affiliations:** 1 Department of Electrical and Computer Engineering, McGill University, 3480 University Street,Montreal, Canada H3A 2A7; E-Mails: mohamad.hajjhassan@mail.mcgill.ca (M. H.); sam.musallam@mcgill.ca (S. M.); 2 Department of Physiology, McGill University, 3655 Promenade Osler, Montreal, Canada H3G 1Y6

**Keywords:** Neural probes, Microfabrication, Biocompatibility, Microelectrodes, Brain machine interfaces, Neural prosthesis, NeuroMEMS, BioMEMS

## Abstract

Neural probe technologies have already had a significant positive effect on our understanding of the brain by revealing the functioning of networks of biological neurons. Probes are implanted in different areas of the brain to record and/or stimulate specific sites in the brain. Neural probes are currently used in many clinical settings for diagnosis of brain diseases such as seizers, epilepsy, migraine, Alzheimer's, and dementia. We find these devices assisting paralyzed patients by allowing them to operate computers or robots using their neural activity. In recent years, probe technologies were assisted by rapid advancements in microfabrication and microelectronic technologies and thus are enabling highly functional and robust neural probes which are opening new and exciting avenues in neural sciences and brain machine interfaces. With a wide variety of probes that have been designed, fabricated, and tested to date, this review aims to provide an overview of the advances and recent progress in the microfabrication techniques of neural probes. In addition, we aim to highlight the challenges faced in developing and implementing ultra-long multi-site recording probes that are needed to monitor neural activity from deeper regions in the brain. Finally, we review techniques that can improve the biocompatibility of the neural probes to minimize the immune response and encourage neural growth around the electrodes for long term implantation studies.

## 1. Introduction

The first documented use of electrical current as a strategic approach to overcome a neural disease (paralysis) can be traced back to the year 1757 [1]. Many of the early research initiatives that investigated the possibility of using electrical signals to cure neural diseases can be found in the review by McNeal [2]. In the year 1939, Hodgkin and Huxley first demonstrated the recording of action potentials from inside a nerve fiber [3]. Again in 1952, the group analytically described the mechanism of nerve excitation and the subsequent generation of action potentials [4, 5]. Over the past several years, neural electrodes or probes became a key tool to record the action potentials from neurons [6], stimulate specific brain regions [7-14], and ultimately understand the functioning of the brain. The stimulation and recording from a single neuron [15-17] or complex networks of neurons using multiple electrodes [18-22] in cortical and sensory areas in brain [23-25] help in understanding various neural characteristics such as population encoding [26, 27], somatosensory organization [28-30], nervous system behavior [31-33], and network connectivity [20].

To date, a wide range of neural electrodes have been used in basic neuroscience and neural prosthetic research (brain machine interfaces) starting with the early electrolyte-filled micropipettes [6, 34-37] and later metal electrodes to the current emerging MicroElectroMechanical Systems (MEMS) based electrodes and polymer electrodes. Since 1950s, single wire metal microelectrodes have been commonly used to record electrical activity in the extracellular environments. These electrodes consist of an insulated metal wire except for the wire tip which represents the recording site [38, 39]. Multiple metal electrode arrays have been used that are made of wire bundles [40], gluing individual metal wire electrodes together [41, 42] or assembling several metal wires on a ceramic plate [43]. During 1970s, Wise and Angell took advantage of the developments in thin-film and integrated circuit microfabrication techniques and reported pioneering work on the first silicon-based microprobes to interface neural tissues [44]. Since then, widespread use of silicon micromachining techniques to develop miniaturized neural probes and probe arrays have led to the establishment of the field of Neural MEMS or NeuroMEMS [44-50]. More recently, polymeric microprobes have received a great deal of attention owing to their simple fabrication process, flexibility and biocompatibility [51-57]. For instance, polyimide based neural probes have been developed that are flexible and mechanically resistant with integrated electrodes on one or both sides of the shank [54] and others have been reported with integrated microtank and microchannels for targeted drug delivery [51]. More recently, some research groups are aiming to develop hybrid neural probes which have the combined capability to record electrical activity as well as specific neural biochemical markers [58]. These new hybrid probes could provide new foresights into complex brain diseases.

A number of reviews have been previously published for neural probes and neuroMEMS by Najafi [12], Banks [48], Heiduschka and Thanos [13], Stieglitz and Meyer [14], and more recently, Donoghue [59], Wise [60] and Pearce and Justin [49]. In particular, these reviews describe generic methods to interface probes with neural tissue, neural probe design and structure, integration of probes with Integrated Circuit (ICs), and biocompatibility issues. In the current review, we specifically focus on the microfabrication techniques and processes for developing the neural probes. We notice that many of the fabrication techniques aim towards cost effective, simple, high yield, ultra-long (high aspect ratio), monolithic integration with microelectronics, and scalable manufacturing. This review will cover three common types of neural probes that are based on metal wire, silicon, and polymer. The next Section details the emerging trends and needs for neural probes. In Section 3, we describe the fabrication of metal probes and the techniques used to expose the recording area while insulating the rest of the probe. In Section 4, silicon based neural probes fabricated with standard microfabrication (MEMS) technology are discussed. In Section 5, we review the polymer based probes which have gained a recent attention due to their biocompatibility and better malleability. Finally, in Section 6 we discuss biocompatibility issues that are related to all functional implantable neural probes. We discuss techniques to improve the biocompatibility of the neural probes in order achieve long-term implantation by improving the ability of the probe to interface with a neural tissue without provoking a natural immune response which deteriorates the probe characteristics.

## 2. Background for Neural Probes

Neural probes are microstructures that form the connection between the biological neural tissue with physical devices and electronics. Implantable neural probes for neuroscience and brain machine interfaces are generally preferred to have a minimum footprint as possible to minimize neural damage and incorporate structural features that facilitate easy entry and movement through the brain tissue. The smaller footprint for the neural probe will allow the probe to approach the target neurons as closely as possible and thus improving the signal-to-noise ratio of the recorded activity or target a very specific region to be stimulated. Further, there is growing need to insert large numbers of accurately spaced recording sites into small volumes which is promoting the application of thin-film and micromachining techniques to the microelectrode production.

Typically, a MEMS-based neural probe consists of a single or multiple long protruding structures or shafts (of lengths ranging from 200 μm to 15 mm) preferably with thin thickness (thicknesses ranging from 10 μm to 200 μm). Each shaft includes the recording sites, interconnect traces and back carrier area carrying the bonding pads to connect the probes to electronics. Ideally, shafts with minimum thickness are preferred to reduce damage to the tissue when the probe is inserted into the brain. Moreover, miniaturization of the neural probes is very crucial to approach and efficiently interface the neural tissue at the scales of neural cells whose size typically range between 10 and 50 μm in diameter and spaced 20 nm apart [61]. In contrast, the shaft has to be wide enough to hold many recording sites to measure neural electrical activity at different depths and route interconnect traces to connect the recording sites to the bonding pads. In addition, the probes should incorporate sufficient mechanical strength to survive the compression and tension forces during the insertion and retraction phases respectively while implantation.

Many research groups investigating neural probe technologies are faced with different challenges including non-standard and unconventional fabrication processes leading to low yield and high cost, lack of on-site and monolithic Integrated Circuit (IC) integration leading to high noise and reduced sensitivity, shorter sized probes mainly limited by fabrication technology, low design flexibility, and limited selection of materials having the mechanical properties that fulfill both the implantation application requirements and being compatible with standard microfabrication processing. In addition, biocompatibility of the neural probes to minimize the foreign-body immune response plays an important role in determining long-term function of neural probes during implantation. These parameters in combination ultimately determine the microfabrication process needed to manufacture the neural probes.

## 3. Metal Wire Based Neural Probes

Metal microelectrodes, which enabled the initiation of many pioneering studies in neural sciences, represent the most widely used neural probes [11]. The electrodes typically consist of electrolytically sharpened wires that are commonly less than 100 μm in diameter and are completely insulated except for a small exposed area at the tip which forms the recording or stimulation site. The electrodes have a tapered structure to enable convenient insertion in the brain tissue. The tapered structure is achieved by dipping the electrodes into an etching solution and slowly drawing out the electrode from the solution. For example, a tungsten wire can be tapered using a sodium hydroxide (NaOH) solution [62]. By varying the composition of the etching solution and speed with which the wire is drawn from the etchant one can control the taper of the electrode.

Typically used electrode materials include platinum, iridium, platinum-iridium, gold, stainless steel, tungsten, and molybdenum. Quartz glass, Teflon, polyimide, and Parylene materials are used for the insulation of these electrodes. The insulating materials ideally should have a high dielectric constant in order to minimize stray capacitances affecting the electrode during recording. A multiple-electrode array of these insulated wires can be made by gluing individual metal wire electrodes together or by using cutoff wire bundles. For instance, Vassiliy Tsytsarev *et al.* [42] have developed a new 8×8 planar array of metal electrodes for in vivo multisite extracellular recording from a rat auditory cortex. The electrode array was formed by alternately gluing, with epoxy, insulated platinum wire electrodes and insulated copper wire spacers. The array of wires was covered by polyethylene tubing and then inserted into stainless steel tubing. The electrode array was connected to the commercial MED64 system from Panasonic which is comprised of an integrated amplifier, connector, and software for data acquisition and analysis.

**Figure 1.**
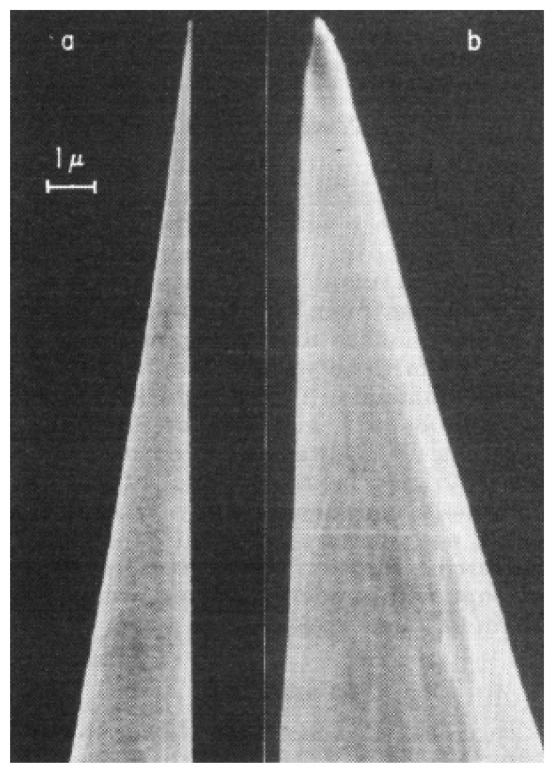
Scanning electron micrographs of microelectrodes made with: (a) DC electropolishing technique and (b) Standard AC electroetching. Reprinted from [39] © IEEE.

The exposed or non-insulated area of metal wire electrodes which forms the recording site is further processed for manipulating the impedance of the site. For instance, Skrzypek and Keller covered a tungsten rod with polymethyl methacrylate (PMMA) and placed it in a Scanning Electron Microscope (SEM) such that the desired area on the probe tip was exposed by manual control of the SEM electron beam. The sample probes are shown in Figure 1 [39]. The degraded PMMA was removed using a solution of ethyl alcohol.

**Figure 2.**
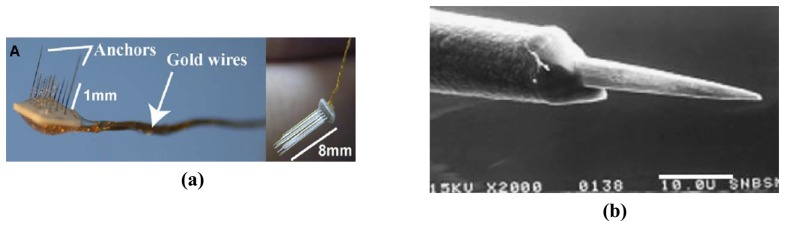
**(a)** Floating multi-electrode array with short and long electrodes. Reprinted from [43] with permission from Elsevier; **(b)** An SEM micrograph of an electrode used in the floating multi-electrode array (FMA) pretreated with A-174 Silane before being insulated with 3mμ of Parylene-C. The tip was exposed by a non-thermal ablation technique using a dual beam Eximer laser system. Reprinted from [63], with permission from Elsevier.

More recently, Musallam *et al.* has reported a floating array of metal probe by employing laser machining of a ceramic substrate to form recording holes [43]. The laser drilled holes are 90 μm wide to accommodate electrodes having a diameter of 80 μm. The probe wires are then coated with Parylene-C for insulation and the wire tips are exposed using a dual beam Eximer laser. The fabricated floating array is shown in Figure 2a. The use of Eximer laser to etch Parylene-C was reported by Schmidt *et al.* as shown in Figure 2b [63].

Although metal based electrodes are still employed successfully in both acute and chronic recording from the brain tissue, these kinds of electrodes can record activity only at their exposed tip. Increasing the number of recording sites requires increasing the number of electrodes which results in a linear increase in overall probe size and causes undesirable neural tissue damage. Further, it is difficult to insert a large number of probes accurately into a small volume of brain tissue. The main advantage of metal electrodes is the simplicity in their manufacturing. This simplicity also leads to its disadvantage which is the lack of common standards and automation in electrode manufacturing from one institution to other. Due to the lack of automation, the electrical characteristics of the recording tip vary from one microelectrode to the next within a batch, from batch to batch, and from one laboratory to other.

**Figure 3.**
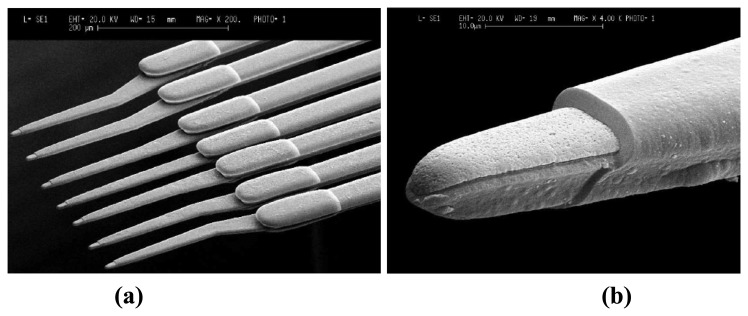
SEM Micrographs of: **(a)** Seven array microelectrode tip regions. **(b)** The Parylene electrode in a Parylene window on the tip. Reprinted from [64], with permission from Elsevier.

**Figure 4.**
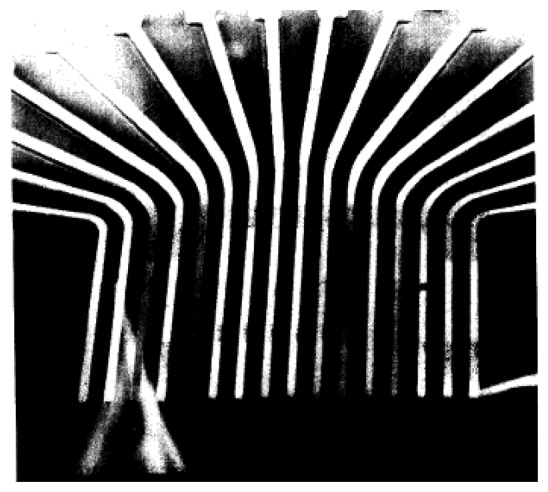
SEM micrograph of the microelectrode array. The Probes are 25 μm wide, 15 μm thick, and 1.15 mm long and have a probe-to-probe spacing of 75 μm. Reprinted from [65]; © IEEE.

The micromachining of metal wire based neural probe arrays has been demonstrated using electroplating technique as it provides high aspect ratio structures for various metals. This process consists of fabricating multiple metal shafts on the same silicon substrate and then releasing them by back etching of the silicon. The electrodes with an insulation layer, consisting of Parylene-C as shown in Figure 3 [64] or silicon nitride as shown in Figure 4 [65], is applied and is followed by photolithographic patterning to expose the recording areas. Hiroaki Oka *et al.* [66] employed electroplating in developing a planar multielectrode array, known as the MED probe, to perform in-vitro electrophysiological studies on acute hippocampal slices. The array consists of 64 platinum black microelectrodes electroplated on a transparent Pyrex substrate. Layers of nickel and gold were non-electrically plated to form bonding pads. Indium-tin oxide was selected for its transparency to form interconnects between the platinum electrode and the bonding pads. Finally, the array was insulated with polyimide except for the platinum electrodes and the bonding pads. Recently, electroplating was used to produce tapered shanks in a single plating step [67, 68]. The use of a patterned seed layer enables creation of 3-D structures with varying thickness and the process was first reported by Maciossek [69].

**Figure 5.**
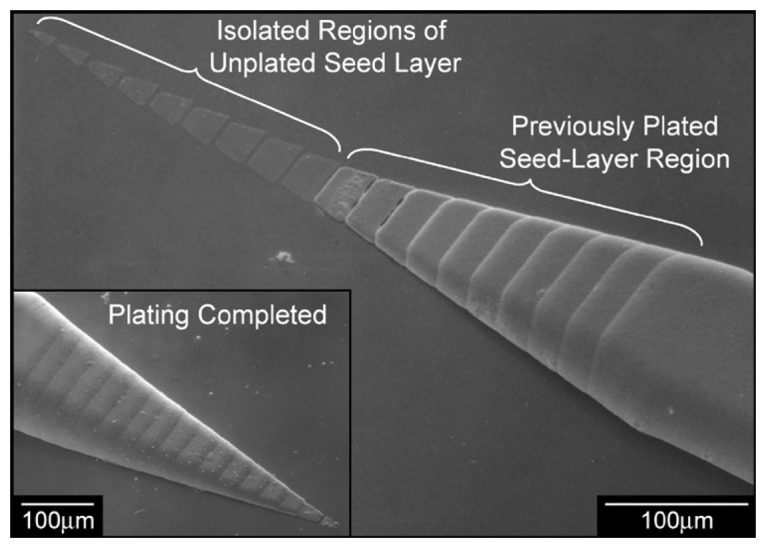
Electrodeposition of the mechanical layer in the probe shank. The SEM image shows the seed-layer regions that are not plated and those previously plated. The inset image on the low left shows the finished tip of the electroplated microprobe. Reprinted from [68]; © IEEE.

The fabrication starts with patterning multiple regions of seed layer with all except one layer being electrically isolated from the electroplating. The electroplated nickel which was used in the process will grow upward and outward from the edges of the patterned seed layer and results in a structure with rounded edges both in- and out- of plane as shown in Figure 5.

During electrodeposition as the height of the electroplated metal equals the height of the neighboring electrically isolated region of the seed layer, plating will occur even on the insulated regions. The electroplating technique is mainly limited by anisotropic deposition rate due to the current crowding effect. Specifically, the electric field lines leave the cathode uniformly and crowd on the exposed metal regions on the wafer.

The result is a higher deposition rate laterally than vertically. Moreover, wire-bonding cannot be used to connect the probes to external circuitry so wires have to be manually soldered to the probe bond pads. The silicon based probes discussed in the next section appear to be more attractive option than metal probes as each can carry many recording sites that are placed at relatively known positions on the probe. Thus, the increase in the number of the recording sites does not increase the overall probe size.

## 4. Silicon Based Neural Probes

The introduction of surface and bulk micromachining techniques played a significant role in standardizing the production of microprobes with very well defined shanks and precise placement of recording sites [70, 71]. The high accuracy and repeatability inherent in these techniques have overcome the problems associated with manual (non-automated) microelectrode fabrication. Moreover, batch processing can be utilized to mass-produce the microprobes at very low cost. Using silicon micromachining one can obtain an active probe with on-probe signal processing circuitry and/or integrated micro actuators driving the electrode shank in order to track the neuron movement [72-79]. The fabrication of silicon based neural probes includes deposition of a metal layer on an insulated substrate and patterning the metal layer to form recording sites, read-out pads for connecting to external circuitry, and interconnecting traces between the recording sites and read-out pads. An insulating layer is then deposited over the whole structure and patterned to open access above the recording sites and bonding pads.

The substrate forms an important part of the probe structure providing the mechanical support. Ideally, the substrate must be biocompatible, small enough to obviate damage to the tissue and strong enough to penetrate the *pia arachnoid* membrane covering the brain. Silicon has well-recognized mechanical advantages suitable for neural probes and it allows the use of the well-established microfabrication technologies and equipment infrastructure developed for the integrated circuits industry leading to high precision and well-defined structural features. The use of silicon photolithography process allow excellent control over the recording site size, shape and spacing enabling multiple recording sites to be placed at variable heights on a single electrode shank. Such ability allows insertion of a large number of recording sites in a small volume which is not possible with metal wire arrays or bundles. Inclusion of integrated circuits can be performed directly on the probes and offers benefits in terms of better signal acquisition, reducing power line interference, and ultimately reducing the overall probe size by reducing the electrical cable sizes [73-83].

**Figure 6.**
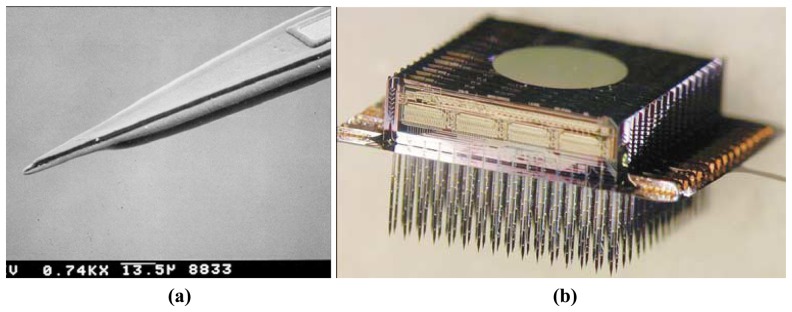
**(a)** Scanning electron microscopy side view of a silicon probe substrate defined using a shallow (tip) and deep (shank) boron etch-step (above) and perspective view (below). **(b)** Photo of a 3-D 1024-site 128-channel neuroelectronic interface. Reprinted from [84]; © IEEE.

A well-known example of silicon based neural probes are the Michigan probes [84] and an example of which is shown in Figure 6. The groups led by Wise and Najafi have developed a variety of neural electrodes including single-shaft, multi-shaft, and 3-D-stacked layouts [11, 44, 70, 73-91]. In addition, many of the developed electrodes are integrated with microelectronic circuitry for signal processing. The fabrication process for these probes typically involved anisotropic etching with ethylene diamine pyrocatechol (EDP) and using a boron-etch-stop. The process is based on the fact that the etch rate for p-type silicon is much slower than for un-doped silicon. Boron diffusion is first performed on silicon to define the shaft shape in the substrate and followed by EDP wet etching to release the probe shafts by having a rounded cross-section and a rounded sharpened tip. Gold, platinum, or iridium metal is used for recording sites. The insulation on top of silicon substrate is made with triple layers of silicon dioxide, silicon nitride, and silicon dioxide. Finally, the interconnection is made with a 4–5 μm thick polysilicon cables which are reported as weak cables and easy to break leading to lower yield for long lengths because of the high aspect ratio and lack of robustness [84, 91, 92].

**Figure 7.**
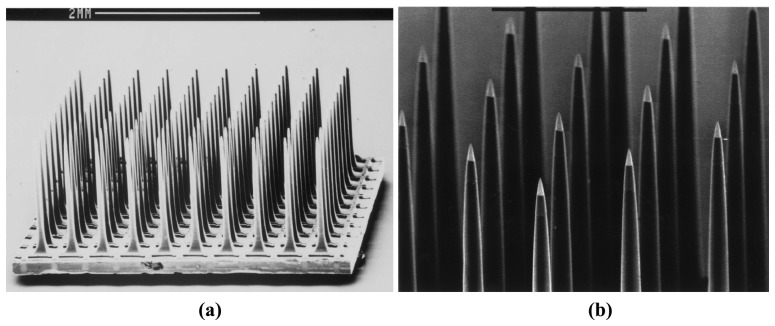
SEM micrograph of: **(a)** The Utah array. **(b)** The insulation coated electrodes with exposed platinum tips. Reprinted from [93], with permission from Elsevier.

Michigan probes have been successfully used in a number of neuroscience applications, but they also suffer from some disadvantages related to probe thickness and durability. Typically, wet etching can be employed for limited probe thickness and thus we find that the typical thickness of the Michigan probes is 15 μm. These probes when pierced through the *pia* layer of the brain needed special guide tools for insertion. Mechanical weakness of the probes causes the probes to crack and shatter and may cause severe damage and disturbance to the brain tissue during insertion. Another well-known silicon based-probes incorporating multiple-electrode arrays is the Utah probe and an example of which is shown in Figure 7 [93-100]. The Utah electrode arrays are typically made from 1.83mm thick boron doped silicon substrates (resistivity of 0.01 Ω)-cm;. A diamond dicing saw is used to create a grid pattern of 300μm deep grooves on the surface of the substrate. A sealing glass is deposited on the grid to create insulation between the electrode bases. Electrode columns are made by sawing a grid on the other side of the silicon and separated by the insulating glass layer. Acid etching smoothes the pillars and creates sharpened probe tips which are then coated with metal for recording and stimulation. Gold, platinum, and iridium are the commonly used metals for the recording purpose. Polyimide is used to coat the probes as the insulation layer with only the recording sites exposed.

The Utah electrode arrays have a unique structure with the arrays pointing upwards (vertically oriented) as opposed to all other silicon neural probes which are built lying down (horizontally oriented). As a result, the probe length of the Utah electrode arrays is limited by the silicon wafer thickness. The longest reported probe length is only 1.5 mm. Further, only one recording site can be made on each probe shaft and the fabrication process is not a typical batch process and therefore suffers from low production rates. The interconnection of the Utah electrode arrays is made of a set of polyimide insulated gold wires and ultrasonically bonded on the back of the array to a set of aluminum read-out pads. The stiffness of the metal wire bundle makes these unsuitable for chronic implantation in human brain and especially for the high-density electrode arrays.

### 4.1. Silicon on Insulator (SOI) based Neural Probes

SOI wafers are produced by placing a thin, insulating layer such as silicon oxide (SiO_2_) or glass sandwiched between a thin layer of silicon (device layer) and the silicon substrate (handle wafer). SOI based neural probes employ the SiO_2_ layer as an etch-stop layer. Several etching techniques are available where the etch rates for SiO_2_ are much lower than for silicon. The handle wafer is back-etched to release the probe shaft which means that the probe shaft thickness is defined by the thickness of the device layer. Different techniques have been used to develop neural probes using SOI wafers, starting with the use of backside wet-etching using potassium hydroxide (KOH) [101] solutions. Plasma etching technology for silicon was also employed which led to the establishment of new fabrication processes to develop neural probes. Plasma etching is a physical-chemical dry etching technique which offers several advantages over traditional wet etching including more reliability and yields a smoother and cleaner etched surface. The plasma etching also has other advantages including relative insensitivity of the etch rate for silicon to its electrical conductivity, less corrosion problems for metal features in the recording sites, and less undercutting and broadening of the photoresist features. Therefore, the use of SOI wafers combined with plasma etching technique provides a good control over the final probe thickness compared to using wet etching with a boron etch-stop. The SOI neural probes are made using CMOS-compatible batch process with multiple electrode sites on each probe shaft. Probes with different thicknesses can also be made by choosing SOI wafers with different device layer thicknesses [101, 102].

**Figure 8.**
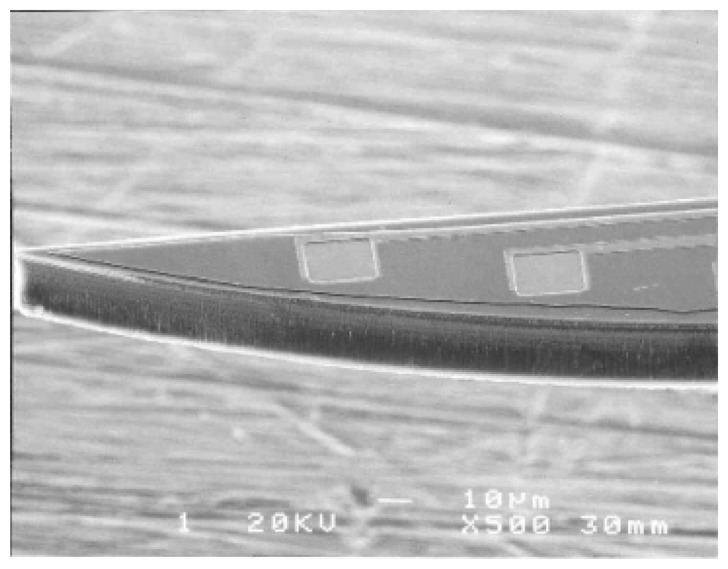
Electrodes at the tip of each probe are separated by 45 μm spacing. Each electrode is 20μm x 20μm square. Reprinted from [83]; © (2003) IEEE.

**Figure 9.**
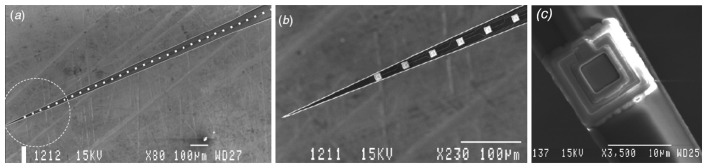
Scanning electron micrograph of: **(a)** A silicon probe with 1 shaft × 32 electrodes. **(b)** Close-up of a probe tip designed with 4° taper angle. The interconnect lines shown are 1μm wide. **(c)** Close-up of a 10 μm × 10 μm Ir electrode site. Reprinted from [102], with permission from Institute of Physics Publishing.

A combination of plasma etching (to shape the probe shafts) with KOH wet etching of the handle wafer (to release the shafts) was used by Kewley *et al.* [103]. Another combination of SOI wet etching, to embed microchannel in the device layer, and plasma dry etching to release the probe shaft was also reported [83]. An improved technology to fabricate silicon neural probes using the Deep Reactive Ion Etching (DRIE) in SOI wafer to overcome the use of wet etching and boron etch stop was developed by Cheung *et al.* and these fabricated probes are shown in Figure 8 [83, 104]. Also, Norlin *et al.* used double-sided DRIE of a SOI substrate with the buried SiO_2_ layer acting as an etch stop to pattern forklike probe shafts as shown in Figure 9 [102].

**Figure 10.**
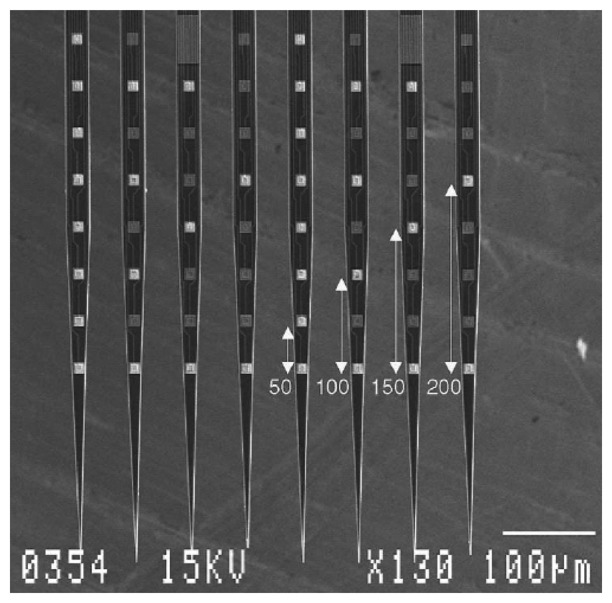
Scanning electron micrograph showing selectively opened electrode windows in the Si_3_N_4_ layer on a 64-site probe. Bright squares are opened windows. Distances shown are in micrometers. Reprinted from [105], with permission from Elsevier.

More recently, Kindlundh *et al.* used DRIE in combination with direct write laser lithography (DWL) for neural probe fabrication [105]. DWL is used to replace the photolithography technique with fixed lithographic mask sets and the process is relatively straightforward but is suitable mostly for small production volumes. The fabricated sample is shown in Figure 10.

### 4.2. Standard Commercial MEMS Process based probes

There are many emerging MEMS and CMOS-MEMS processes that can be used to fabricate neural probes with specific features such as ultra-long reinforced structures and integrated signal processing capability. Typically, we find micromachined electrode arrays are constructed using silicon as the structural support layer with gold as the recording sites material. These materials are available in the standard MEMS processes such as SOIMUMPS from MEMSCAP and MicraGEM from Micralyne. Further, the use of standard fabrication processes yields mass-producible and well-defined probe structures. More recently, CMOS-MEMS process from DALSA 0.8 μm process with bulk micromachining can be employed for development of active and intelligent neural electrodes. However, post-fabrication processing is required for the CMOS-MEMS process to deposit gold or iridium on the electrode recording sites.

The authors recently developed prototype neural electrodes using the MicraGEM process. The prototype includes high-aspect ratio silicon electrodes which have 10 μm thickness and up to 6.5 mm in length, although longer probes are possible. Ultra-long probes having lengths longer than 5 mm can be used for recording in deeper regions of the brain. Many probes developed previously for this purpose by several research groups failed (cracked or shattered) during implantation as they were unable to withstand the insertion axial forces, retraction forces, and tension forces of the brain tissue. To overcome the problem, the developed prototype investigates a novel principle of reinforcing the probes in their more susceptible areas to improve their strength during implantation without increasing the dimensions of the probe.

**Figure 11.**
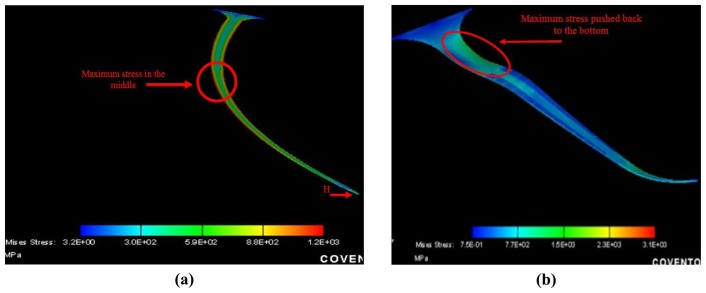
Behavior of the 6.5mm long and 10 μm thick electrode when it is subjected to horizontal shear force: **(a)** Without reinforcement. **(b)** With reinforcement.

**Figure 12.**
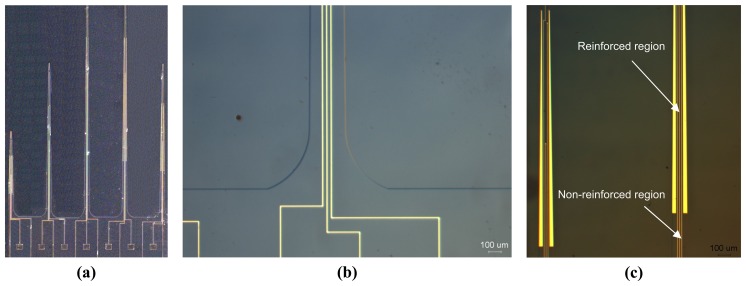
Multi-electrode probe array fabricated by using the MicraGEM process from Micralyne. **(a)** The multi-length array. **(b)** Tapered base of the probe. **(c)** Probe Reinforcement.

Numerical studies are performed to determine the reliability of the ultra-long neural probes when subjected to significant shear stresses due to imperfect insertion into the brain and/or due to movement of the brain relative to the skull. The simulations are used to predict the behaviour of the probes when subjected to a buckling force under axial compression. The horizontal component of the force is very important for neural electrodes as it determines the force at which the probe tip penetrates the brain tissue. As seen from the simulation results in Figure 11a, the maximum stress for the 6.5 mm long probe (thickness is 10 μm and width is 75 μm) is in the middle where the probe is the most susceptible for breakage. Therefore, reinforcing the middle of the probe with additional metal layers would lead to increased strength. We notice that adding a strip of metal to the probe increased its tensile strength by 10% when compared to the standard probe by pushing back the maximum stress in the middle to the base of the probe as shown in Figure 11b. This enables the probe to be more resistant to the shear force exerted by the brain tissue surface during the insertion phase.The fabricated sample is shown in Figure 12a. Figure 12b and Figure 12c compare the two probes without and with the metal layer reinforcement.

## 5. Polymer based neural probes

The use of polymer materials for neural probes has been widely studied to replace the normal silicon nitride or silicon dioxide insulation layer deposited during the fabrication of silicon neural electrodes [64, 106-108]. Different biocompatible polymers such as polyimide [106] and Parylene-C are used to cover the metal and silicon region of the probes to form a biocompatible interface between the probe and the brain tissue. For example, polyimide was used to coat the Utah electrodes arrays [94, 95]. Parylene-C was used as insulating layer on silicon probes [64]. More recently, polymers on metal probes was used wherein each probe encompasses a single recording site and a dual beam Eximer Laser system process was used to open the probe recording tips [43]. Polymer materials are also used for the interconnect cables. For example, microfabricated polyimide cables [109] and PDMS cables [110] are reported as interconnections for the silicon neural probes. However, unconventional bonding methods are still required to connect the polymer cables with the microfabricated silicon probes.

**Figure 13.**
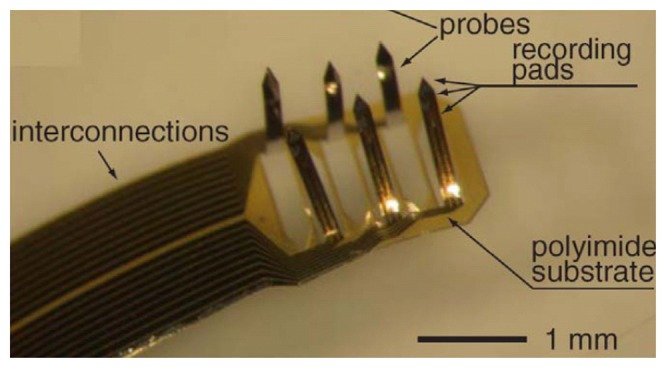
3D flexible probe array after folding. The recording pads are vertically aligned. Reprinted from [111], with permission from Institute of Physics Publishing.

**Figure 14.**
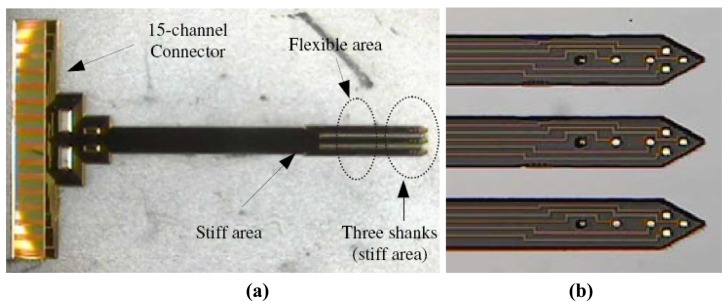
Optical microscope images of the polyimide neural probes **(a)** View of the electrode. **(b)** Top view of the three shanks. Reprinted from [112], with permission from Institute of Physics Publishing.

Rousche *et al.* [51] demonstrated a flexible polyimide neural probe wherein a gold metal layer was used for the recording sites. The interconnection traces and read-out pads are sandwiched between two polyimide layers. Similar devices were also reported by Shoji Takeuchi *et al.* [111] as shown in Figure 13. 3-D probes can be formed by physically bending the polyimide shanks out of the 2-D plane. The use of polyimide improves the malleability match between a rigid electrode and soft tissue and the lack of which results in tissue damage if the electrode moves relative to brain. A major drawback to this design is that the electrodes are not stiff enough to pierce brain tissue on their own, so implant sites had to be created with metal wire or a scalpel before insertion. An improved polyimide probe was reported by Lee *et al.* [112] and is shown in Figure 14, where a 5-10 μm thin silicon support layer formed from silicon-on insulator (SOI) substrate is attached to the desired region of the probe shaft to increase the stiffness. The stiffness of the electrode can be varied by changing the thickness of the silicon backbone layer. A similar polyimide probe with 15 μm molybdenum backbone was developed by Blum *et al.* [113].

The flexible polyimide electrodes suffer from disadvantages due to the lack of rigidity which causes buckling during insertion into the brain. Given this reason, long length polyimide probes cannot be used as they bend due to lack of rigidity and reduces the accuracy in targeting a selected region in the brain. Moreover, polyimide-based probes are prone to failure due to possible moisture absorption by polyimide. Given this, silicon-based neural probes appear to be more successful owing to the mechanical properties of the silicon and the flexibility of patterning using standard microfabrication technologies.

## 6. Biocompatibility of Neural Probes

Perhaps the greatest challenge facing the use of neural implants is the resultant tissue response to the neural injury which effects the long term functioning of the implanted neural probe. Signal deterioration soon after implantation of arrays has been reported by many groups. For these devices to be useful, they should ideally remain functional indefinitely for enabling self-contained and self-governing brain machine interfaces. Signal deterioration has been attributed to the general immune activation of the brain in response to the presence of a foreign device. Insertion of the electrode ruptures the vasculature and destroys all neurons in its path. Immediately following the insertion of an array, an acute immune response is activated which facilitates the recruitment of glial cells [114]. Activated glial cells will commence digesting the cellular debris with enzymes. The extent of the response is a function of probe size [114], probe shape [115] and surface texture [116]. This initial response gives way to the chronic response where scarring in the brain leads to the beginning of probe encapsulation [114].

Using immunohistology, Biran *et al.* [117] showed that the glial density around implanted probes increases with time, further isolating the electrodes from neurons. Further damage is elicited by the motion of the electrode within the brain [118]. The brain experiences micromotion in response to physiological processes such as cardiac rhythms [119] and head movements [120]. This motion may cause the electrodes to rip the neural tissue as the stiffness of the brain and the electrodes differ. Formation of the glial scar needs to be minimized in order to extend the lifetime of the arrays. Two strategies that are currently being investigated include methods to minimize the immune response or the addition of proteins to encourage neural growth. Injection of dexamethasone postoperatively to attenuate inflammation has been shown to decrease the initial and long term response [121]. Local delivery of dexamethasone at the site of injury may prove to be a more effective method since delivery can be sustained indefinitely [122]. Anti-inflammatory agents have been used to coat the electrode [123]. This method has also been shown to be effective in reducing glial response.

In contrast to directly battling the immune response, some groups have proposed using neural growth factors to encourage neural growth around the electrode. Kennedy used a glass cone filled with growth factor releasing tissue and showed that using neural growth factors (NGF) improves recording over many months [124]. In addition, Moxon *et al.* recently showed that using nanostructured porous silicon (PSi) surface for implants improved biocompatibility [125]. They reported decreased astrocyte adhesion on nanoporous silicon without any interference in the electrode's ability to record the action potentials [108, 126-128].

## 7. Conclusions

The paper aimed to give an overview of the commonly used microfabrication technologies to develop neural probes for prosthesis and brain machine interfaces. Technological advancements in the areas of microelectronics along with surface and bulk micromachining have led to the development of a great number of passive and active probes. In the review, we described metal wire, silicon, and polymer based neural electrodes. Silicon based probes appear to be the most versatile and widely accepted technology for neural microelectrodes. We notice that there are three main considerations for the development of neural electrodes, namely, (i) probe material selection, (ii) microfabrication process, and (iii) biocompatibility interface. The combination of these three factors ultimately determines the functionality, usability, and long-term implantation reliability of the neural electrodes.

Implantable neural probes are generally preferred to have a minimum footprint as possible to minimize neural damage and to facilitate easy entry and movement through the brain tissue. The above requirement combined with the growing interest to undertake neuroscience studies from deeper regions of the brain has necessitated the need to develop ultra-long probes (lengths longer than 5mm) with thicknesses less than 50μm. Such high aspect ratio structures pose a great design challenge as the probes have to be able to withstand the insertion axial forces, retraction forces, and tension forces of the brain tissue. New techniques such as probe reinforcement as described in this review have to be developed for these emerging applications. In near future, the standardization of commercial MEMS processes and emerging CMOS-MEMS processes could lead to the development of new neural probes that are cost-effective and mass-produced with ultra-long probe shafts and on-site/on-probe signal processing circuitry.
